# Precise genome-wide base editing by the CRISPR Nickase system in yeast

**DOI:** 10.1038/s41598-017-02013-7

**Published:** 2017-05-18

**Authors:** Atsushi Satomura, Ryosuke Nishioka, Hitoshi Mori, Kosuke Sato, Kouichi Kuroda, Mitsuyoshi Ueda

**Affiliations:** 1 Division of Applied Life Sciences, Graduate School of Agriculture, Kyoto University, Sakyo-ku, Kyoto Japan; 2 Japan Society for the Promotion of Science, Sakyo-ku, Kyoto, Japan

## Abstract

The CRISPR/Cas9 system has been applied to efficient genome editing in many eukaryotic cells. However, the bases that can be edited by this system have been limited to those within the protospacer adjacent motif (PAM) and guide RNA-targeting sequences. In this study, we developed a genome-wide base editing technology, “CRISPR Nickase system” that utilizes a single Cas9 nickase. This system was free from the limitation of editable bases that was observed in the CRISPR/Cas9 system, and was able to precisely edit bases up to 53 bp from the nicking site. In addition, this system showed no off-target editing, in contrast to the CRISPR/Cas9 system. Coupling the CRISPR Nickase system with yeast gap repair cloning enabled the construction of yeast mutants within only five days. The CRISPR Nickase system provides a versatile and powerful technology for rapid, site-specific, and precise base editing in yeast.

## Introduction

The budding yeast *Saccharomyces cerevisiae* has been a central eukaryotic model cell for many decades. After the genome sequence of *S. cerevisiae* was determined, many mutations have been introduced into the yeast genome to examine the functions of the genes and to regulate protein functions^1–4^. As the reverse genetic techniques, some marker genes such as auxotrophic markers were used for targeted genomic mutagenesis^5^. However, these techniques cannot rule out the possibility that the marker genes influence the phenotypes. For the marker-less technique, a two-step integration/excision method has been used in yeast^6,7^. This method relies on a selection step based on marker gene insertion, and a subsequent marker-removing step by counter-selection. However, the integration/excision method is inefficient and time-consuming in that the steps of plasmid construction, integration, and marker-removal take approximately two weeks.

For efficient, marker-less, targeted genomic mutagenesis technology, the clustered regularly interspaced short palindromic repeat (CRISPR)/Cas9 system has been developed^8^. In the current CRISPR/Cas9 system, Cas9 nuclease is targeted to a specific genomic site by a guide RNA (gRNA)^8^. The Cas9/gRNA complex recognizes a targeting site bearing a complementary 20-nt sequence of gRNA, which is closely followed by a protospacer adjacent motif (PAM), typically the bases NGG. This system leads to double strand breaks (DSBs) at 3 base pairs (bp) upstream of the PAM via the RuvC and HNH nuclease domains. DSBs induce cellular death as well as two independent repair pathways: homology-directed repair (HDR) and non-homologous end joining (NHEJ)^9,10^. NHEJ is an error-prone repair pathway that leaves an insertion or deletion at the cleavage site. HDR is a high-fidelity repair pathway dependent on donor DNA. Therefore, target-specific cleavage by this system can stimulate gene knockout through NHEJ and flexible gene modifications through HDR.

**Figure 1:**
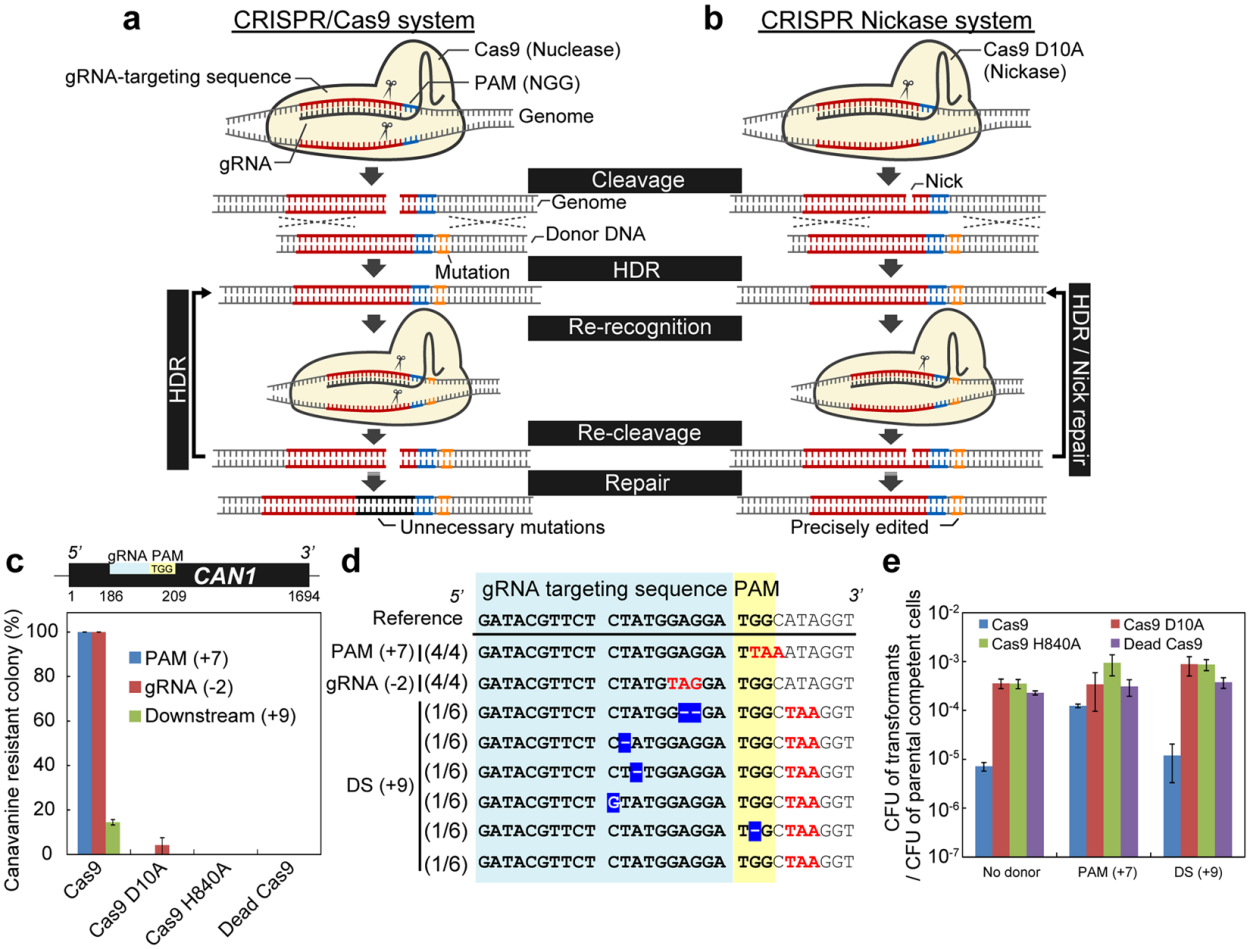
Limitations of the CRISPR/Cas9 system. (**a,b**) Schemes of genome editing at an outside area of the PAM and gRNA-targeting sequences by the CRISPR/Cas9 system (**a**) and by the CRISPR Nickase system (**b**). (**c**) Genome editing efficiencies at the *CAN1* gene by the conventional method^11^. The targeted position is described above the graph. (**d**) Sequencing of the edited *CAN1* gene. Cas9 precisely edited within the PAM and gRNA-targeting sequences, but not at a site downstream (DS) from the cleavage site. Introduced stop codons (red) and unintended mutations (inverted) are indicated. The numbers of observed sequences over the numbers of total sequenced strains (e.g., 4/4) are shown. (**e**) Colony forming efficiencies. The colony forming units (CFUs) on selective medium divided by the CFU of competent cells counted on non-selective medium are presented to evaluate the toxicity of the CRISPR systems. The error bars show standard error of the mean (SEM) based on three independent measurements.

Despite the high efficiency and robustness of the CRISPR/Cas9 system, there are two major problems with this system. First, bases editable through HDR are limited to within the 20-bp gRNA-targeting site and PAM sequence. When mutations are introduced into outside areas of the PAM and gRNA-targeting sequences by HDR, these recognition sequences remain intact even after HDR, which potentially causes re-cleavage by the CRISPR/Cas9 system (Fig. 1a). The re-cleavage will repeatedly induce HDR until the PAM or gRNA-targeting sequence is disrupted by error-prone NHEJ. Therefore, the CRISPR/Cas9 system has been thought to be unable to precisely edit outside areas of the PAM and gRNA-targeting sequences by HDR, while it can precisely edit bases inside these sequences with nearly 100% efficiency in yeast^11^. Second, the specificity of this system is imperfect^8,12,13^. Unintended mutations have sometimes been observed in off-target sites that share sequence homology with the on-target site^14^. For stable and precise genome editing, a new genome editing technology that is capable of genome-wide and target-specific editing needs to be developed.

As a strategy to induce high-fidelity HDR without stimulating NHEJ, the introduction of target-specific single strand breaks (nicks) has drawn attention. Target-specific nicking has been achieved by the Cas9 nickases (Cas9 D10A and Cas9 H840A)^15^. Cas9 D10A cleaves the gRNA-targeting strand, while Cas9 H840A cleaves the non-targeted strand^16^. Nicks have been reported to induce HDR but cause very little NHEJ^17^. However, since HDR efficiency at nicks is generally very low^17,18^, efficient genome editing initiated by nicks has not been realized so far in eukaryotes such as yeast and human cells.

In this study, efficient HDR at nicks stimulated by a single Cas9 nickase was successfully achieved by promoting cell cycles so that yeast cells underwent sufficient S/G2 phases at which HDR is induced^19^. The constructed “CRISPR Nickase system” efficiently edited bases regardless of whether they were inside or outside of the PAM and gRNA-targeting sequences, while the CRISPR/Cas9 system could only edit within these sequences. Moreover, this new system was highly specific to the on-target site, unlike the CRISPR/Cas9 system. Coupling the CRISPR Nickase system with yeast gap repair cloning (GRC) enabled yeast-mutant construction in only 5 days. The CRISPR Nickase system serves as a valuable, versatile, and powerful genomics tool in *S. cerevisiae*.

## Results

### Limitations of the CRISPR/Cas9 system

To investigate whether the CRISPR/Cas9 system can precisely edit the outside areas of recognition sequences, we used a previously reported CRISPR/Cas9 system in *S. cerevisiae* ^11^. Here, a centromeric single-copy plasmid constitutively expresses Cas9 under the control of the strong *TEF1* promoter, and a multi-copy 2 μ plasmid constitutively expresses gRNA from the *snR52* promoter. gRNA was designed to target the *CAN1* gene, which encodes an arginine transporter^20^. Nonsense mutations in the *CAN1* gene confer canavanine resistance to yeast cells, while the functional *CAN1* gene causes canavanine sensitivity. Using canavanine sensitivity and resistance, we evaluated the genome editing efficiencies of the CRISPR/Cas9 system by introducing stop codons in the PAM sequence, gRNA-targeting sequence, and an outside area of the PAM and gRNA-targeting sequences. Oligo nucleotide as a donor DNA (80 bp) was used to mediate the editing of the *CAN1* gene. When the CRISPR/Cas9 system introduced stop codons at the PAM and gRNA-targeting sequences, almost all yeast cells acquired canavanine resistance (Fig. 1c), which was a similar efficiency to that reported previously^11^. A catalytically inactive Cas9 (Dead Cas9) was used as a negative control and it did not induce genome editing. All sequenced colonies obtained intended stop codons in the *CAN1* gene (Fig. 1d). However, when a stop codon was introduced at the outside area (9 bp downstream from the cleavage site) by using Cas9, only a few cells acquired canavanine resistance (Fig. 1c). Furthermore, many cells obtained unintended mutations in the gRNA-targeting sequences as well as the intended premature stop codon (Fig. 1d). Only one colony underwent the intended genome editing. Expressing Cas9 from multi-copy plasmids resulted in similar efficiencies (Fig. S1a,c) and the inaccurate genome editing (Fig. S1b,d). In both cases, the CRISPR/Cas9 system precisely edited the PAM sequence, but not the outside area.

Colony forming efficiencies varied greatly depending on the mutation sites in the donor DNA (Fig. 1e). Cas9 production in the absence of donor DNA severely decreased colony forming efficiency, because DSB induced cellular death as well as NHEJ. Conversely, donor DNA containing the mutation in the PAM sequence protected cells producing Cas9 from cellular death caused by DSB. However, donor DNA containing the mutation at the outside area led to cellular death, suggesting that repeated cleavages by Cas9 occurred, as expected.

### Development of the CRISPR Nickase system

**Figure 2:**
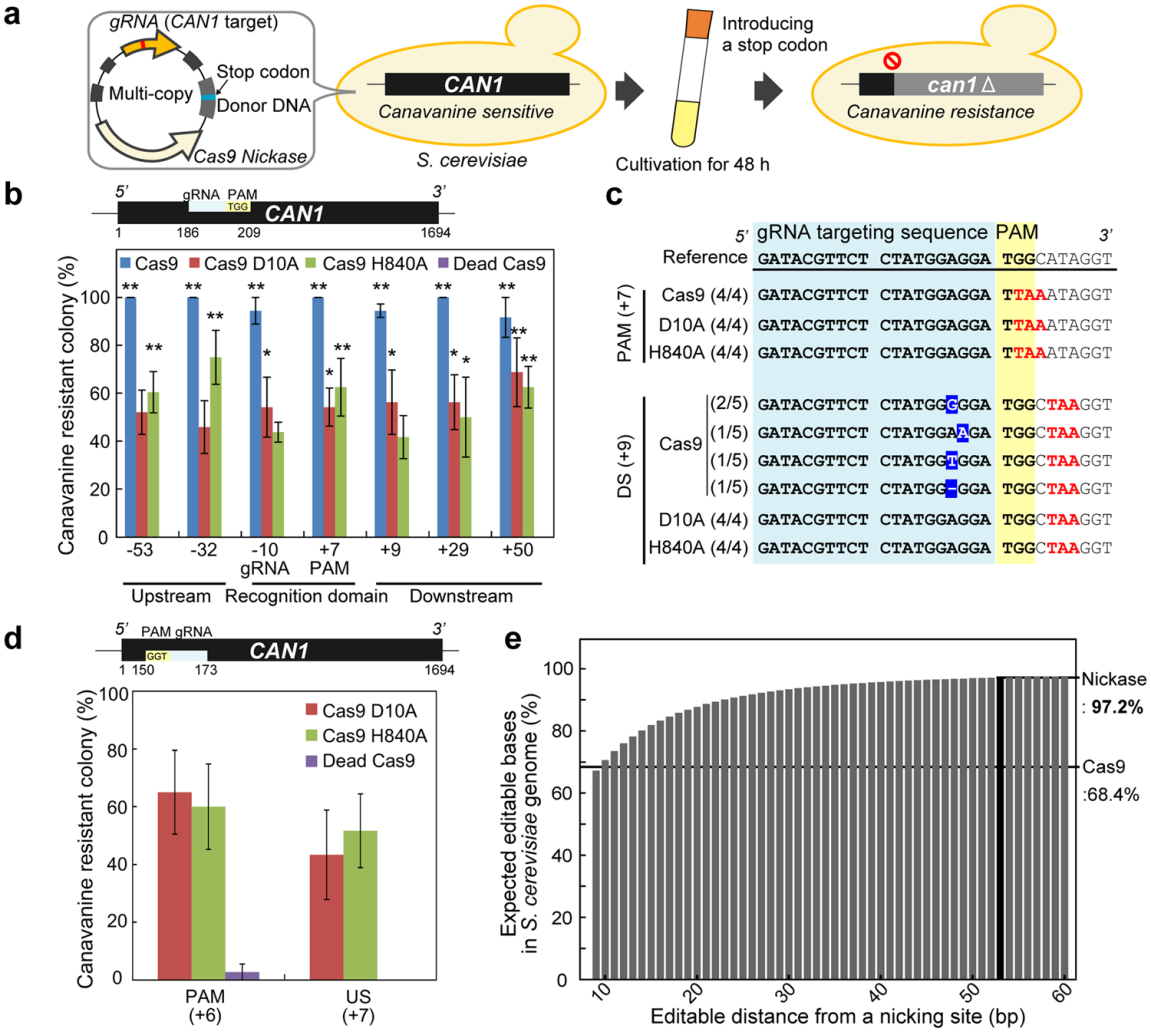
Construction of the CRISPR Nickase system. (**a**) Scheme of the experiment. Cas9 nickase- and gRNA-expressing cassettes were integrated into a single plasmid that contained a donor DNA sequence. Transformants were cultivated in selective medium for 48 h from an OD_600_ of 10^−5^ (approximately 1.1 × 10^3^ cells). (**b**) Genome editing efficiencies at the *CAN1* gene. Efficiencies were calculated based on canavanine assays. The numbers indicate the distances (bp) of edited sites from the cleavage site. (**c**) Sequence analysis. Introduced stop codons (red) and unintended mutations (inverted) are represented. The numbers of observed sequences over the numbers of total sequenced strains are shown. All sequence results corresponding to Fig. 2b are described in Fig. S2. DS, downstream from the cleavage site. (**d**) Editing efficiencies at the different targeting sequence on the opposite strand of the *CAN1* gene. Sequencing is shown in Fig. S3b. (*e*) Theoretical bases editable by the CRISPR Nickase system. The x-axis indicates the hypothetical editable distances from the nicking site. The y-axis shows theoretical editable bases (%) in the *S. cerevisiae* genome. The error bars show SEM based on at least three independent measurements. *P* values were determined by comparing between each sample and dead Cas9 control in each site, based on Tukey’s test. *P < 0.05, **P < 0.01.

Since the CRISPR/Cas9 system had the limitation of the editable bases, we attempted to develop a new genome editing technology that is free from this limitation. To achieve this, we used the Cas9 nickases (Cas9 D10A and Cas9 H840A)^15^, instead of Cas9 nuclease (Fig. 1b). Nickase introduces nicks that can be precisely repaired in cells^21^. A nick can induce homologous recombination, but not NHEJ^15^. Therefore, repeated re-recognition and re-cleavage by the Cas9 nickase/gRNA after HDR would not stimulate NHEJ, resulting in precise genome editing. However, simple replacement of the Cas9 nuclease with Cas9 nickase (Cas9 D10A or Cas9 H840A) did not induce efficient genome editing at all (Fig. 1c), even when Cas9 nickase was produced from multi-copy plasmids (Fig. S1a,c). We thought that transient donor introduction was not enough to stimulate the nick-mediated HDR. Nicks are precisely filled in cells and do not induce HDR as efficiently as DSB^17^. Therefore, we integrated the donor DNA fragment into the plasmid producing the Cas9 nickase and gRNA to prevent losses of donor by degradation and cell division (Fig. 2a). About 1000 bp of donor DNA was used in this study, because more than 700 bp donor length has been reported to promote sufficient HDR in *S. cerevisiae* ^22^. Additionally, transformants that harbored the plasmids were further cultivated in liquid medium for 48 h. Since HDR is induced in the late S and G2 phases when sister chromatids are available as HDR templates^19^, promoting cell cycles by cell division was expected to confer more chances for cells to repair nicks by HDR.

First, we tested this system by producing Cas9 nuclease as a positive control (Fig. 2b). In this vector design, Cas9 production induced efficient and precise genome editing at the PAM sequence, suggesting that this system worked as expected (Fig. 2b,c). When the outside areas were edited, Cas9 introduced the intended stop codons as well as unintended mutations around the cleavage sites (Fig. 2c and Fig. S2). Production of Cas9 without donor DNA or with donor DNA containing mutations at the outside area caused severe decreases in colony forming efficiencies, while donor DNA containing the mutation in the PAM sequence protected cells (Fig. S3a). These results emphasize the fact that prevention of re-recognition by the Cas9/gRNA complex after HDR is crucial for accurate genome editing. When Cas9 nickase was produced to edit the PAM sequence, approximately 50% of cells obtained canavanine resistance and the intended stop codon in the *CAN1* gene (Fig. 2b,c). Notably, Cas9 nickase was able to introduce mutations at the outside areas with approximately 50% efficiency, without any unintended mutations (Fig. 2b,c). Furthermore, this system successfully edited bases up to 53 bp upstream and 50 bp downstream from the nicking site (Fig. 2b). There were not significant biases in efficiencies between the two nickases, Cas9 D10A and Cas9 H840A (*P* = 0.500 according to two-way ANNOVA), indicating that the nicked strand did not affect the HDR efficiency. Colony forming efficiencies of cells producing Cas9 nickases were not varied by mutation sites in the donor DNA (Fig. S3a). Furthermore, next generation sequencing revealed that Cas9 D10A and Cas9 H840A did not introduce any unintended mutations into *S. cerevisiae* genome, at least when PAM and outside area (+9 downstream from the nicking site) were edited. This system could precisely edit bases at different sites in the *CAN1* gene (Fig. 2d and Fig. S3b) and in the different strain (Fig. S3c,d). This system was also able to edit the *LYP1* gene and introduced not only stop codons in the ORF, but also a frame-shifting mutation (Fig. S3e,f). Lyp1p is lysine transporter and the destruction of *LYP1* confers thialysine resistance to the cells^23^. In all cases, genome was precisely edited regardless of the sites with about 50% efficiency. We termed this base editing technology using Cas9 D10A or Cas9 H840A as “CRISPR Nickase system”.

These results suggest that the CRISPR/Cas9 system can only edit the inside bases of the gRNA-targeting sequence (20 bp) and the PAM sequence (NGG; 2 bp). According to our *in silico* calculation, bases editable by the CRISPR/Cas9 system account for 68.4% of the whole *S. cerevisiae* genome (Fig. 2e). The remaining 31.6% were the areas that were not covered by any PAM or potential gRNA-targeting sequences. In contrast, the CRISPR Nickase system, which can precisely edit much broader areas, can theoretically edit 97.2% of the bases in the *S. cerevisiae* genome, according to a similar *in silico* calculation (Fig. 2e). This system overcame the major limitation of the CRISPR/Cas9 system.

### Specificity of the CRISPR Nickase system

**Figure 3:**
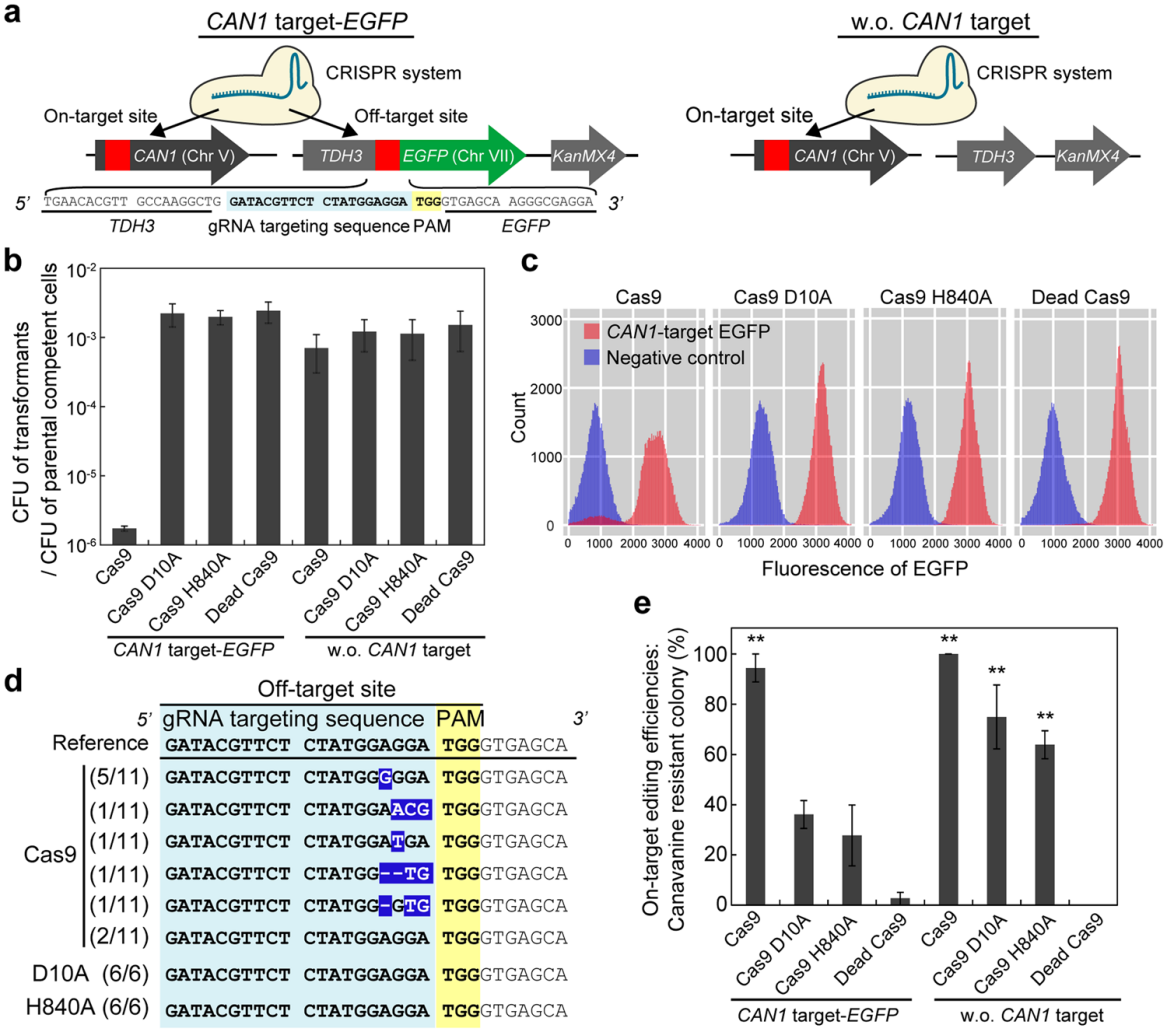
Specificity of the CRISPR Nickase system. (**a**) Scheme of specificity assay. The artificial off-target site and *EGFP* gene were fused to the downstream from the strongly expressed *TDH3* gene (*CAN1*-target EGFP). A strain that did not harbor the off-target site or the *EGFP* gene (w.o. *CAN1* target) was used as a negative control. (**b**) Colony forming efficiencies. The CFUs of transformants on selective medium divided by the CFU of competent cells counted on non-selective medium are presented to evaluate the toxicity of the CRISPR systems. (**c**) Analysis of EGFP fluorescence. Histograms of the fluorescent intensities of the cells are shown. (**d**) Sequencing of the off-target site. Unintended mutations are inverted. The numbers of observed sequences over the numbers of total sequenced strains are shown. (**e**) Genome editing efficiencies at the on-target *CAN1* site. The error bars show SEM based on three independent measurements. *P* values were determined by comparing between each sample and dead Cas9 control in each strain, based on Tukey’s test. *P < 0.05, **P < 0.01.

The CRISPR/Cas9 system has been reported to induce unintended mutations at off-target sites that have sequence similarities with the on-target site^12,14,24^. On the other hand, the CRISPR Nickase system was not expected to induce off-target editing, because induction of nicks at off-target sites is unlikely to cause NHEJ^25^. In order to investigate the specificity of the CRISPR/Cas9 and CRISPR Nickase system, we integrated an artificial off-target site at upstream from an *EGFP* gene. The off-target site had the same sequence as the on-target site in the endogenous *CAN1* gene (Fig. 3a). Since the on-target site in the *CAN1* gene was repaired through HDR using donor DNA containing the mutation in the PAM sequence, Cas9 and Cas9-nickase production did not cause cellular death in the absence of the off-target site (Fig. 3b). In the presence of the off-target site, however, the colony forming efficiency of Cas9 producing cells was severely decreased due to DSB at the off-target site, while that of cells producing either nickase was not decreased. This fact indicates that the CRISPR Nickase system did not induce cellular toxicity and DSBs at the off-target site. Fluorescence analysis revealed that some cells producing Cas9 lost fluorescence (Fig. 3c and Fig. S4b). Unintended mutations were found at the off-target site even in the rest of fluorescent cells (Fig. 3d). In *S. cerevisiae*, not all cells underwent frame-shifting mutations, when Cas9 was produced to cleave the *CAN1* gene without donor DNA (Fig. S4c,d). In contrast, the CRISPR Nickase system induced neither loss of fluorescence nor unintended mutations at the nicking site (Fig. 3c,d). Furthermore, this system edited the on-target site without any errors (Fig. 3e and Fig. S4e). These results suggest that the CRISPR Nickase system can distinguish on-target and off-target sites even if both sequences are identical.

### Rapid construction of mutants

**Figure 4:**
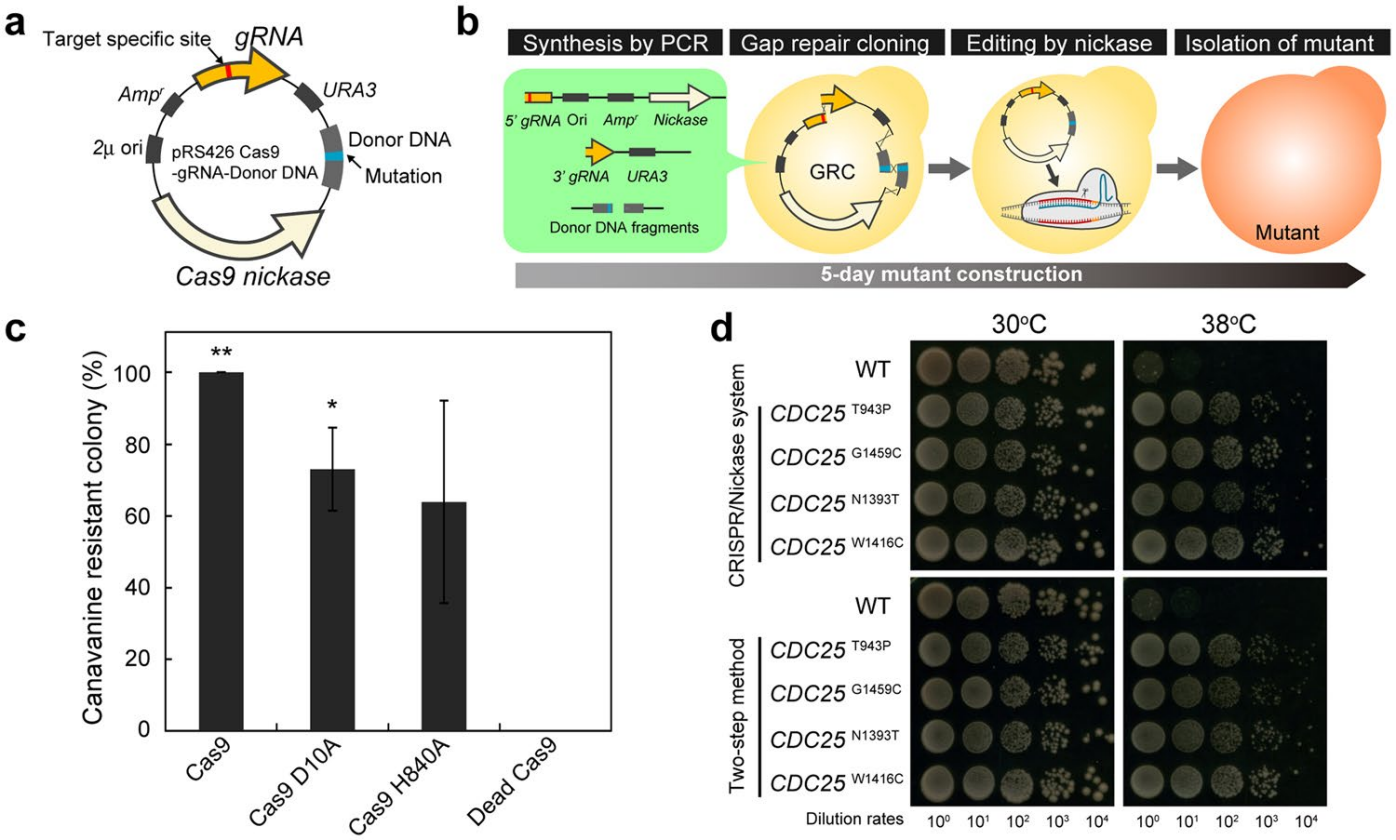
The CRISPR Nickase system coupled with GRC. (**a**) Plasmid design for the CRISPR Nickase system. (**b**) Scheme of the CRISPR Nickase system coupled with GRC to construct mutants in five days. (**c**) Efficiencies of genome editing at the *CAN1* gene coupled with GRC. The error bars show SEM based on three independent measurements. (**d**) Thermotolerance of *CDC25* mutants constructed by the CRISPR Nickase system coupled with GRC. Cells (OD_600_ = 1.0) were diluted 10, 10^2^, 10^3^, and 10^4^ fold and spotted onto YPD plates. Since major differences in efficiencies between Cas9 D10A and Cas9 H840A had not been observed, Cas9 D10A was used in this experiment. The *CDC25* mutants constructed via the CRISPR Nickase system and a conventional two-step integration/excision method are compared. *P* values were determined by comparing between each sample and dead Cas9 control, based on Tukey’s test. *P < 0.05, **P < 0.01.

The CRISPR Nickase system was revealed to have advantages for genome-wide base editing and specificity over the CRISPR/Cas9 system. This system, however, still had the difficulty for the rapid construction of mutants. In this system, custom-made plasmids bearing donor DNA and gRNA expression cassettes have to be constructed based on the target site, which usually takes about one week (Fig. 4a). In order to achieve the rapid construction of mutants, we combined yeast gap repair cloning (GRC) with the CRISPR Nickase system (Fig. 4b). GRC is a genetic technique that depends on the ability of yeast to assemble DNA fragments to construct plasmids in the cells^26^. It only requires short overlapping sequences at the ends of the fragments that can be easily added by PCR. Parts of plasmid fragments were amplified to attach a target-specific gRNA sequence and mutated sequence, as well as to attach overlapping sequences to both ends of each fragment, and directly introduced into yeast cells (Fig. S5a). We assembled cassettes expressing the Cas9, Cas9 D10A, Cas9 H840A, or Dead Cas9 as well as a gRNA-expressing cassette, and donor DNA by GRC to introduce stop codons at the PAM sequence of the *CAN1* gene. The CRISPR/Cas9 system as well as the CRISPR Nickase system coupled with GRC achieved precise genome editing at the *CAN1* gene in only five days (Fig. 4c and S5b). In addition to the *can1*Δ mutant, we constructed *CDC25* mutants to demonstrate the capability of this system with GRC. Cdc25p is a membrane bound guanine nucleotide exchange factor, and mutations in this protein lead to thermotolerance in yeast^27^. Importantly, the CRISPR Nickase system coupled with GRC precisely introduced mutations regardless of whether they were inside (W1416C) or outside (T943P, G1459C, and N1393T) of the PAM and gRNA-targeting sequences (Fig. S5c). The *CDC25* mutants constructed by this system exhibited thermotolerance as expected (Fig. 4d). These phenotypes did not differ from those of *CDC25* mutants constructed by the conventional two-step insertion/excision technique (Fig. 4d).

### Investigation of key proteins for nick-repair via HDR

**Figure 5:**
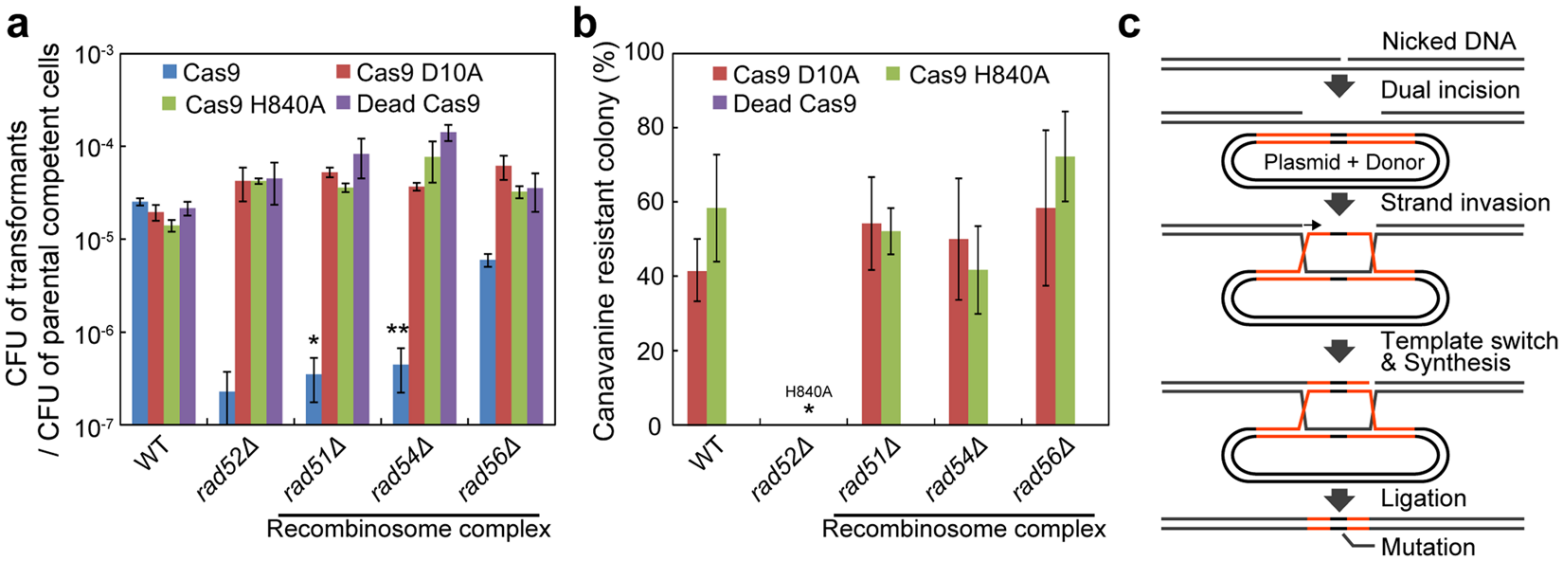
Essential proteins for the repair of nicks via HDR. (**a**) Colony forming efficiencies in *rad-*deleted strains. The targeting site for Cas9, Cas9 D10A, Cas9 H840A, and dead Cas9 was the same as in Fig. 2b. The donor DNA in the plasmids contained a stop codon in the PAM sequence. The CFUs of transformants on selective medium divided by the CFU of competent cells counted on non-selective medium are presented to evaluate which Rad-proteins were required for the repairs of DSBs and nicks. *P* values were determined by comparing between each sample and dead Cas9 control in each strain, based on Tukey’s test. (**b**) Genome editing efficiencies at the *CAN1* gene by the CRISPR Nickase system in *rad*-deleted strains. *P* values were determined by comparing between each sample and WT strain expressing Cas9 D10A or Cas9 H840A, based on Tukey’s test. *P < 0.05, **P < 0.01. (**c**) Working model of HDR at single strand breaks. The error bars show SEM based on more than three independent measurements.

The key proteins for the repair of nicks via HDR have not been elucidated in *S. cerevisiae*. Rad51p and Rad52p play important roles in repair of DSBs via HDR^28^. Consistent with this fact, Cas9 production in each *rad51* and *rad52* knockout mutant strain greatly inhibited colony formation even in the presence of donor DNA (Fig. 5a). In the *rad52*Δ strain, the CRISPR Nickase system could not induce genome editing at all, suggesting that Rad52p plays critical roles in repair of nicks via HDR (Fig. 5b). This fact indicates that promotion of cell cycles in the liquid medium enhanced genome editing efficiencies by providing the opportunity with cells to undergo the S/G2 phases at which *RAD52* is expressed^29^. On the other hand, the CRISPR Nickase system was able to induce genome editing in the absence of Rad51p. Supporting this, this system could induce genome editing without Rad54p and Rad56p, which function with Rad51p as the “recombinosome complex” (Fig. 5b)^28^. These results strongly suggest that repair of single strand breaks via HDR is dependent on Rad52p but is independent of Rad51p.

## Discussion

In this study, we successfully developed genome-wide base editing technology, CRISPR Nickase system. This system was able to edit bases at sites where the conventional CRISPR/Cas9 system was unable to edit. When outside areas of the PAM and gRNA-targeting sequences were edited using oligonucleotides as donor DNA by the CRISPR/Cas9 system, unintended mutations were found at various sites in the recognition sequences (Fig. 1d, Fig. S1b,d). These mutations were possibly derived from mutations randomly present in the oligonucleotides. On the other hand, when donor DNA resided in a plasmid, unintended mutations were found only around the cleavage site (Fig. S2). Since the mutation patterns were very similar to that of NHEJ (Fig. S4b), repeated cleavage would finally induce NHEJ in the cells. In both cases, the CRISPR/Cas9 system failed to precisely edit the outside areas, limiting the utility of this system. One possible way to avoid repeated cleavages may be to induce Cas9 expression only transiently to prevent prolonged Cas9 cleavages. However, mutation frequencies achieved by the temporary expression of Cas9 were less than 0.1%^11^, probably because transient induction would not provide enough Cas9 protein for efficient genome editing. A previous report suggested that Cas9 abundance in cells was the one of the most important factors determining editing efficiency^30^. Using donor DNA containing silent mutations in the recognition sequences will be able to avoid unintended mutations in the cleavage sites. However, this approach limits the editable sites only within ORF. In addition, there have been some reports which described that codons not only encode amino-acid sequences, but also regulate gene expression levels by functioning as enhancers^31,32^. Therefore, while silent mutation can maintain the information of amino-acid sequences, it potentially disrupts regulatory sequences in the ORF. For efficient genome-wide base editing, transcription activator-like effector nuclease (TALEN) technology would be suitable^33,34^. Since TALEN technology does not require any motif sequences like the PAM sequence, TALEN can be constructed to target all areas in the genome, in contrast to the CRISPR/Cas9 system. However, unlike the CRISPR/Cas9 system, TALEN needs correct alignment of binding modules, which requires time-consuming DNA manipulations^25,26^. Although the use of other types of CRISPR-related systems with different PAM requirements will expand the editable bases, the utilization of different systems based on the targeting-sites is laborious and renders the system low throughput^35^. To introduce intended single-point mutations, a genetic technique dependent on the expression of single strand DNA (ssDNA) *in vivo* has been developed in *Escherichia coli* ^36^. This technique relies on the target-specific mutagenic property of mutated ssDNA. However, mutation efficiencies of ssDNA were very low (less than 2%) in *E. coli* ^36^ and *S. cerevisiae* ^37^. As a similar approach to the CRISPR Nickase system, a double nicking strategy using the Cas9 nickases^38^ has been developed. This strategy uses paired Cas9 nickases that introduce nicks in each DNA strand, causing site-specific DSBs. This technique is highly specific because DSBs occur only when two nickases work simultaneously. However, at least one recognition sequence should be altered after HDR not to induce unwanted mutations at the cleavage site, which limits editable bases. In contrast, the CRISPR Nickase system easily and rapidly edited the PAM, gRNA-targeting sequences, and the outside areas of these sequences in a precise manner. This system is a robust and versatile tool for genome-wide base editing.

The usage of small primer-sized donor DNA will make the CRISPR Nickase system further useful and versatile. Previous study showed that HDR efficiencies between long donor DNA with more than 600 bp homology arms and short donor DNA with 45 bp arms were similar in *S. cerevisiae* ^22^. This result indicates that integration of small donor DNA into the plasmid may not reduce the genome editing efficiency of the CRISPR/Nickase system.

We revealed that nick repair via HDR was independent of Rad51p, although HDR at DSB was profoundly dependent on Rad51p (Fig. 5a)^28^. Little is known about the mechanisms of HDR at nicks in human cells, and even in *S. cerevisiae*. A previous study showed that transcription-coupled repair (TCR) played a partial role in HDR at nicks in human cells^39^. TCR preferentially detects and repairs damage on the transcribed DNA strand^40^. The study also showed that the inhibition of Rad51p enhanced the TCR efficiency. However, any biases in the efficiencies between Cas9 D10A and Cas9 H840A were not observed in this study. Moreover, our study revealed that Rad51p was not involved at all in HDR at nicks. There will be differences in the nick repair mechanisms via HDR between human and *S. cerevisiae* cells. In the absence of Rad51p, break-induced replication (BIR) has been reported to be able to repair DSBs using homologous donor DNA^41^. BIR is a recombination-dependent mechanism which is initiated by DNA synthesis^42^. In BIR, however, site-specific local recombination has not been observed. Instead, the repair process continues to the end of the template DNA, typically up to telomere domains when the donor is chromosomal DNA^41^. Nevertheless, in our study, integrations of the plasmid backbone were not found. This fact suggests that BIR was not involved in HDR at nicks in *S. cerevisiae*. We consider that post-replication repair (PRR)^43^ or a similar mechanism would play a role in HDR at nicks (Fig. 5c). In this model, the first driving force of the recombination is a dual incision, which is observed in nucleotide excision repair^44^. Subsequently, the intact single strand DNA invades the undamaged donor DNA, which was carried on the plasmid in this study. The nicked strand switches the template and extends the strand according to the donor DNA sequence, as is observed in PRR^43^. Consequently, the nick induces genome editing. Supporting this hypothesis, PRR has been reported to be conducted in the absence of Rad51p, but inhibited in the absence of Rad52p^45^.

The CRISPR/Cas9 system has been reported to be sensitive to mismatches in the region close to the PAM (called the seed sequence, typically 1–12 bp from PAM), while being tolerant of mismatches in the distal site from PAM (13–20 bp from PAM)^8^. This suggests that if the CRISPR/Cas9 system introduced single-base substitutions in the gRNA-targeting sequence around the distal site from PAM, re-cleavage will still occur, and unwanted mutations might be observed due to NHEJ. Therefore, this system may be able to robustly edit only the seed sequences as well as PAM sequences. According to our calculation, it means that the coverage of this system would be narrowed down to only 56.0% in the *S. cerevisiae* genome. In addition, a previous study reported that the CRISPR/Cas9 system sometimes non-specifically edited off-target sites that share sequence similarity to the on-target site^14^. This fact suggests that some targeting sites are not suitable for precise editing, which further narrows down the editable areas of the CRISPR/Cas9 system. On the other hand, the CRISPR Nickase system can specifically edit only the on-target site, and apparently does not have any limitations in the targeting sites (Fig. 3).

In the human genome, 69.1% of regions are possible editable areas by the CRISPR/Cas9 system, according to our *in silico* calculation. If editable bases are limited to the seed and PAM sequences, this system covers only 57.6% of the human genome. This indicates that approximately 30.9–42.4% of areas are out of the reach of the CRISPR/Cas9 system. Therefore, the CRISPR Nickase system should also be applied in human cells as a basic genetics tool as well as for clinical use. In human cells, nicks have been reported to induce HDR^39^. HDR activity in human cells, however, is generally low, and NHEJ has been dominantly observed^46^. To enhance HDR, cell cycles will have to be synchronized, because cell cycle largely governs the choice of the two repair pathways, HDR and NHEJ. Cell cycle synchronization at the G2/M phase by nocodazole treatment was reported to improve HDR efficiencies in human cells^47^. Some reagents such as SCR7, L755507, and Brefeldin A have also been reported to enhance the HDR efficiency^48,49^. Additionally, the intracellular abundance of Cas9 protein is also important for efficient editing^30^. However, too much abundance of Cas9 nuclease should not be used to avoid off-target editing and cellular toxicity. The CRISPR Nickase system, on the other hand, apparently did not show any cellular toxicity, while the CRISPR/Cas9 system showed severe toxicity (Fig. 1e, Fig. 3b, and Fig. S3a). Furthermore, since the CRISPR Nickase system induced high fidelity recombination even in the presence of the off-target site (Fig. 3), off-target editing will not be a serious problem in human cells. Therefore, high-level production of Cas9 nickase might be able to achieve efficient genome editing without inducing off-target effects and cellular toxicity.

In this study, we successfully developed a novel genome-wide base editing system. To our knowledge, this is the first approach to specifically edit the outside areas of the PAM and gRNA-targeting sequences. In the case of editing the genomic areas that are not covered by PAM or gRNA-targeting sequences, this system may become the first choice for genome editing. Moreover, this is one of the fastest genetic techniques for inducing genome editing in yeast. Considering the editable areas, high-fidelity, and rapidity of mutant construction, the CRISPR Nickase system will be a robust tool that overcomes the limitations of the CRISPR/Cas9 system.

## Online Methods

### Strains and DNA manipulation

*S. cerevisiae* MT8-1 (*MAT* **a**, *ade*, *his3*, *leu2*, *trp1*, *ura3*)^50^ and BY4741 (*MAT* **a**, *his3*, *leu2*, *met15, ura3*; EUROSCARF, Frankfurt, Germany) were used as host strains for genome editing. The *rad* ^−^ knockout strains (parent strain: BY4741) were obtained from the yeast gene knockout collection^51^. *Escherichia coli* strain DH5α [F^−^, *end*A1, *hsd*R17(r_k_^−^/m_K_^+^), *sup*E44, *thi-*1, λ^−^, *deo*R, *rec*A1, *gyr*A96, *pho*A, ϕ80d*lac*ZΔM15, Δ(*lac*ZYA-*arg*F)U169] (Toyobo, Osaka, Japan) was used as a host for the recombinant DNA manipulation. The primers used to construct plasmids are listed in Table S1. Donor fragments and 20-nt gRNA expressing DNA were inserted into backbone vectors by In-Fusion HD Cloning Kit (Clontech, CA, USA) and Ligation high (Toyobo), respectively. *E. coli* transformants were grown in Luria–Bertani medium [1% (w/v) tryptone, 0.5% (w/v) yeast extract, and 1% (w/v) sodium chloride] containing 50 μg/mL ampicillin. Introductions of one-point mutations by the conventional two-step integration/excision method were conducted using the pAUR135 vector (Takara Bio, Shiga, Japan) following the manufacturer’s instructions.

### Next generation sequencing

Next generation sequencing was performed as previously described^27^. The average depth of each genome analysis was 50.6. Quality of paired-end reads were checked with FastQC (http://www.bioinformatics.babraham.ac.uk/projects/fastqc/) and mapped to the *S. cerevisiae* genome (sacCer3) using Burrows-Wheeler Aligner^52^. To identify genomic mutations, Genome Analysis Toolkit^53^ was used.

### Genome editing by the conventional CRISPR Cas9 system

Genome editing was performed as previously reported^11^. Oligonucleotides as a donor DNA was used by annealing 80-nt single strand oligonucleotides (Table S1). Yeast cells were transformed with 250 ng plasmid and 500 pmol donor DNA using the Frozen-EZ yeast transformation II kit (Zymo Research, CA, USA). Transformants on selective medium were replica-plated onto SD plate medium supplemented with 60 mg/L canavanine and appropriate amino acids without arginine.

### Base editing by the CRISPR Nickase system

Transformation was performed as described above. Transformants were selected and grown on SDC + AW [0.67% (w/v) yeast nitrogen base without amino acids, 2% glucose, 2% casamino acid (BD BioSciences, CA, USA), 2.0 × 10^−3^% adenine, and 2.0 × 10^−3^% l-tryptophan] plate medium. The colonies on the selective medium were suspended in distilled water. The suspension was adjusted to an optical density at 600 nm (OD_600_) of 10^−5^ (approximately 1.1 × 10^3^ cells) in 5 mL SDC + AW liquid medium and grown for 48 h at 30 °C. Cultivated cells were spread on YPD [1% (w/v) yeast extract, 2% (w/v) peptone, 2% (w/v) glucose] plate medium. For *CAN1* mutagenesis analyses, colonies on YPD plate medium were replica- plated to SD plate medium supplemented with 60 mg/L canavanine and appropriate amino acids without arginine to evaluate genome editing efficiencies. For *LYP1* mutagenesis analyses, colonies on YPD plate medium were replica-plated to SD plate medium supplemented with 100 mg/L l-thialysine (Sigma-Aldrich, MO, USA) and appropriate amino acids without lysine. For the sequence analyses, colonies were randomly picked and DNA fragments containing a mutation site were directly amplified by PCR, and then analyzed by Sanger sequencing (Eurofins Genomics, Tokyo, Japan). Primers for direct colony PCR were constructed not to amplify the donor DNA contained in the plasmids (Table S1).

### Calculation of editable bases

The yeast genome used for the calculation of editable bases was obtained from the *Saccharomyces genome* database (http://www.yeastgenome.org/). All bases (1.21 × 10^7^ bp) were classified based on whether editable or not. “GG” and “CC” in PAM sequences and the following 20 bp- or 12 bp- gRNA-targetable sequences in the genome were defined as editable. Genome coverage (%) was calculated by dividing the number of editable bases by the total bases in the genome. In a similar manner, editable bases by the CRISPR Nickase system were determined based on the distances from a nicking site. The whole sequence of human genome was used for the calculation of editable bases in human cells by the CRISPR/Cas9 system (http://hgdownload.cse.ucsc.edu/downloads.html).

### Fluorescence analysis

At least 20 colonies on selective plate medium were suspended in distilled water at the same time. The suspension was adjusted to an OD_600_ of 10^−5^ in 5 mL SDC+AW liquid medium and grown for 48 h at 30 °C. Cells were then inoculated into fresh YPD medium, adjusted to an OD_600_ of 0.1, and cultivated at 30 °C for 12 h. The flow cytometry analysis was performed using a JSAN desktop cell sorter (Bay bioscience, Kobe, Japan). The fluorescence of cells was analyzed using AppSan software (Bay bioscience), and was measured with excitation at 488 nm and emission from 512 to 558 nm. A total of 5.0 × 10^4^ cells was recorded per sample. In order to quantify the proportion of cells producing non-functional EGFP, we asked whether the fluorescence intensities were below the threshold. The threshold was determined based on each negative control distribution, and was defined as the median +2 standard deviations of fluorescence intensity. Cells that did not exceed the threshold were regarded as non-fluorescent.

### Gap repair cloning

DNA fragments were amplified to add arms with more than 30 bp homology at the each end (Table S1). For amplification of a Cas9 D10A and gRNA containing fragment, 2 ng template was used. Donor DNA fragments containing the mutation were amplified directly from yeast cells. Amplified DNA fragments were treated with DpnI (Toyobo) for 2 h at 37 °C to degrade template plasmids completely. Purification was performed with QIAquick PCR purification kit (QIAGEN, Hilden, Germany), and 0.2 pmol DNA fragments were used for transformation. Transformants were cultivated as described above. Sequencing of *CDC25* mutants was performed by randomly picking colonies on YPD plate medium.

### Spot assay

For the spot assay on a YPD plate, cells were pre-cultivated in 5 mL of YPD medium at 30 °C for 12 h. Cells were inoculated into 10 mL of YPD, adjusted to an OD_600_ of 0.1, and incubated at 30 °C for 12 h. The OD_600_ of harvested cells was adjusted to 1.0 and diluted 10, 10^2^, 10^3^, and 10^4^ fold with distilled water. On a YPD plate, 5 μL of each sample was spotted and incubated at 30 °C and 38 °C.

## Electronic supplementary material

Supporting Information

Supplementary Table S1
