# Erratum: Precise genome-wide base editing by the CRISPR Nickase system in yeast

**DOI:** 10.1038/s41598-017-09606-2

**Published:** 2017-09-27

**Authors:** Atsushi Satomura, Ryosuke Nishioka, Hitoshi Mori, Kosuke Sato, Kouichi Kuroda, Mitsuyoshi Ueda

**Affiliations:** 1 Division of Applied Life Sciences, Graduate School of Agriculture, Kyoto University, Sakyo-ku, Kyoto Japan; 2 Japan Society for the Promotion of Science, Sakyo-ku, Kyoto Japan

*Scientific Reports* **7**:2095; doi:10.1038/s41598-017-02013-7; Article published online 18 May 2017

Supplementary Table S1 was omitted from the original version of this Article. This has been corrected in the HTML version of the Article; the PDF version was correct at time of publication.

