# Supplementary material for: Precise genome-wide base editing by the CRISPR Nickase system in yeast
Source: Sci Rep. 2017 May 18;7:2095. doi: 10.1038/s41598-017-02013-7 (PMC5437071; doi:10.1038/s41598-017-02013-7)

Scientific Reports

SUPPLEMENTARY INFORMATION

TITLE

Precise genome-wide base editing by the CRISPR Nickase system in yeast

AUTHORS AND AFFILIATIONS

Atsushi Satomura, Ryosuke Nishioka, Hitoshi Mori, Kosuke Sato, Kouichi Kuroda, Mitsuyoshi Ueda

Supplementary Figure S1 Genome editing by multi-copy vectors expressing Cas9.
(**a**) Genome editing efficiencies at the *CAN1* gene. Cas9 and gRNA were produced from independent multi-copy plasmids. (**b**) Sequencing analysis corresponding to (**a**). (**c**) Cas9 and gRNA were produced from a single multi-copy plasmid. (**d**) Sequence analysis corresponding to (**c**). Introduced stop codons (red) and unintended mutations (inverted) are indicated. The numbers of observed sequences over the numbers of total sequenced strains are shown. DS, downstream from the cleavage site.

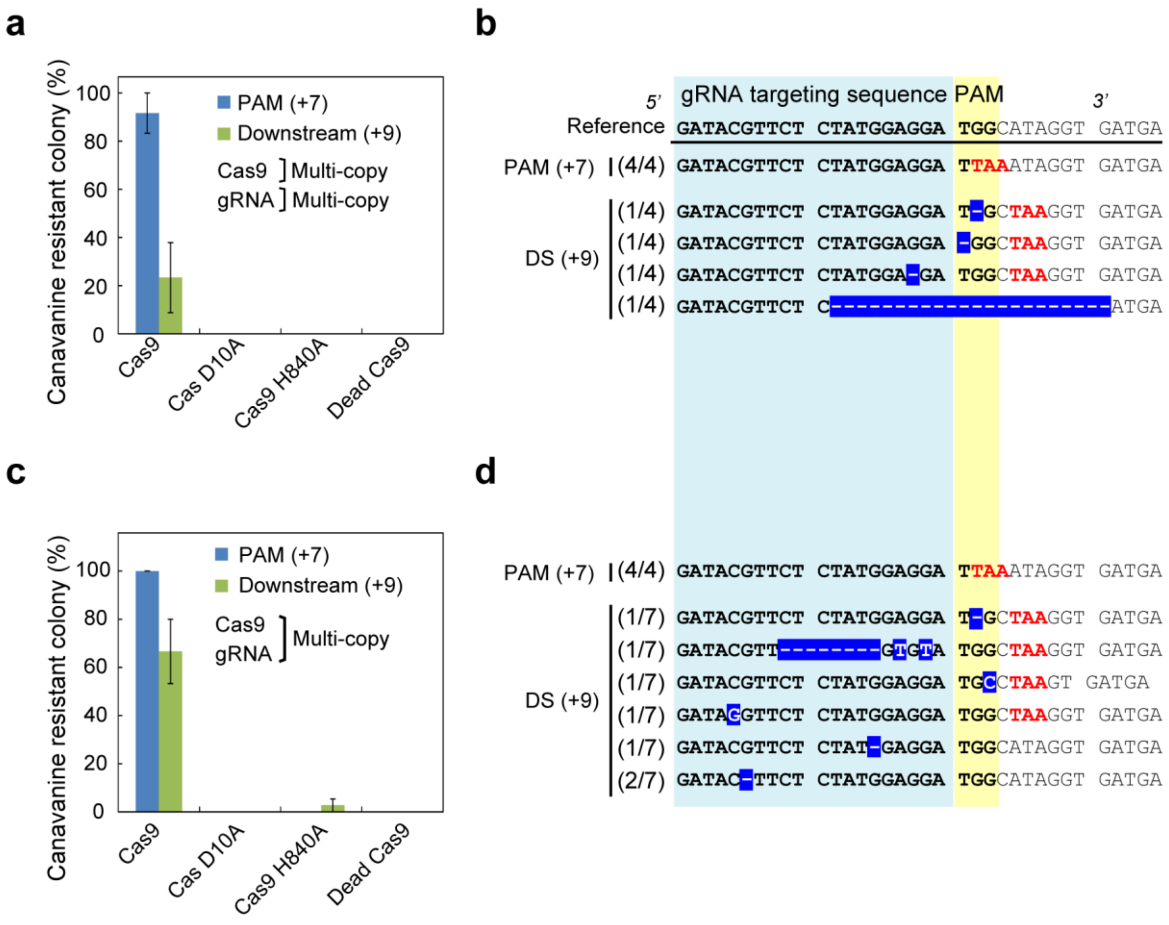

Supplementary Figure S2 Sequence analysis corresponding to Fig. 2b.

Introduced stop codons (red) and unintended mutations (inverted) are shown. The numbers of observed sequences over the numbers of total sequenced strains are shown. DS and US represent downstream and upstream from the cleavage site, respectively.

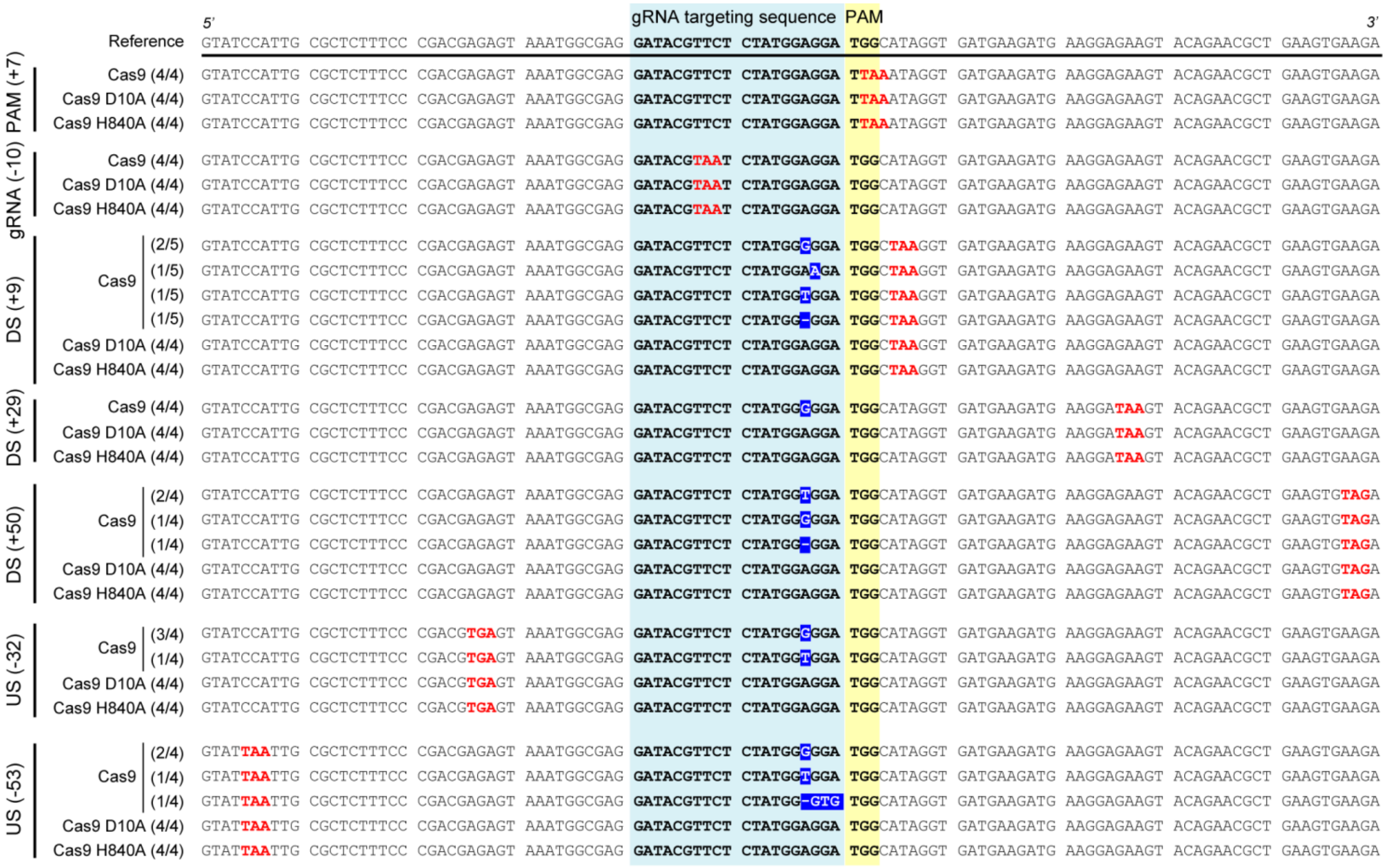

Supplementary Figure S3 Genome editing by the CRISPR/Nickase system.

(a) Colony forming efficiencies. The CFUs of transformants on selective medium divided by the CFU of competent cells counted on non-selective medium are presented to evaluate the toxicity of CRISPR systems. (b, d, f) Sequence analyses corresponding to Fig. 2d, Fig. S3c, and Fig. S3e, respectively. Introduced stop codons and single-base deletions (red) are represented. The numbers of observed sequences over the numbers of total sequenced strains are shown. DS and US represent downstream and upstream from the cleavage sites, respectively. (c) Genome editing efficiencies at the *CAN1* gene. BY4741 was used as a host strain to evaluate the generality of the CRISPR Nickase system. (e) Genome editing efficiencies at the *LYP1* gene. *P* values were determined by comparing between each sample and dead Cas9 control in each site, based on Tukey's test. * $P < 0.05$, ** $P < 0.01$.

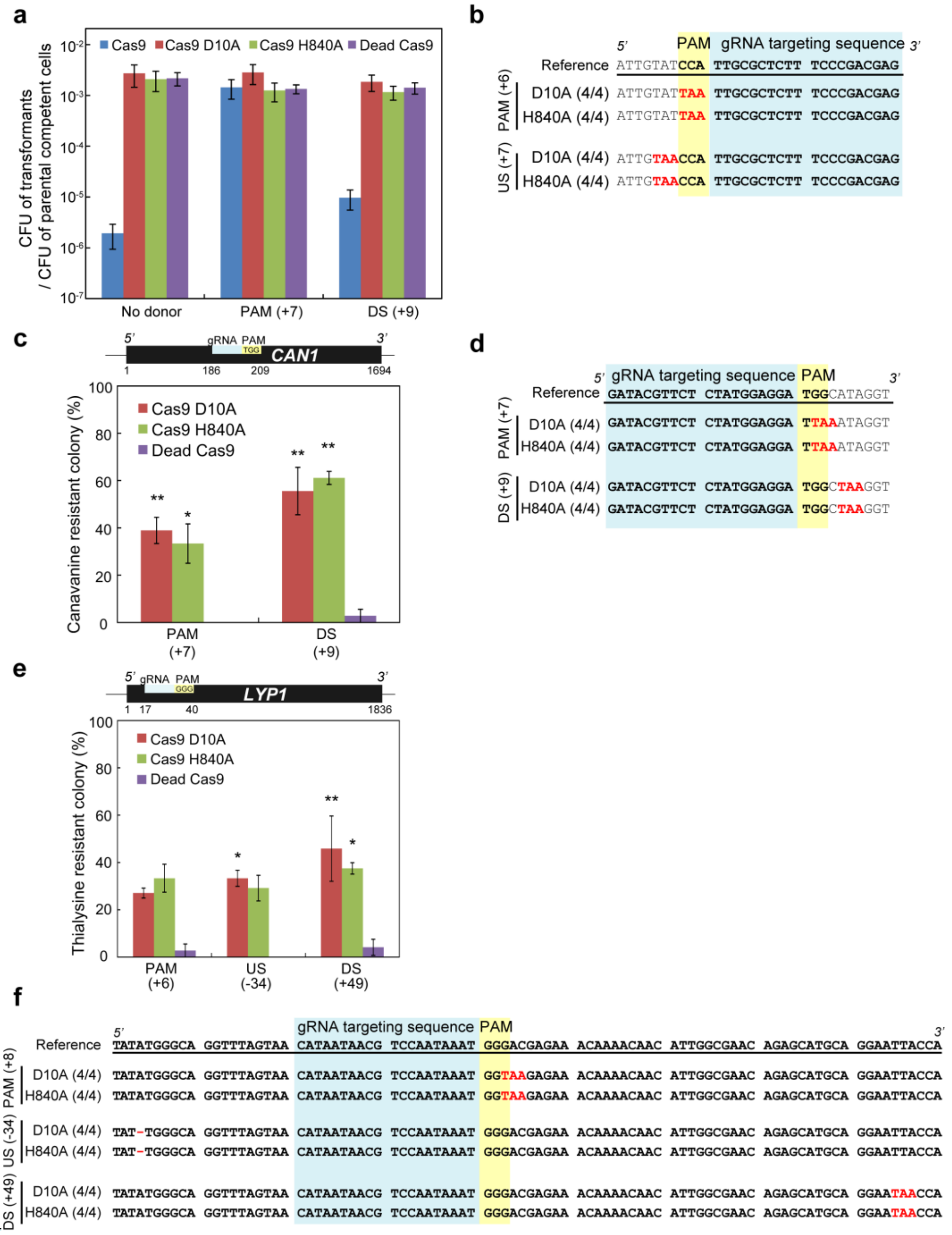

Supplementary Figure S4 Construct of the *CAN1*-target EGFP system.

(a) Fluorescence intensities of *CAN1*-target *EGFP* and w.o. *CAN1* target. (b) Proportion of non-fluorescent cells. (c) Genome editing efficiencies at the *CAN1* gene without donor DNA. Cells acquiring frame-shift mutations through NHEJ were able to obtain canavanine resistance. (d) Sequence analyses. Mutations caused by NHEJ (inverted) are shown. (e) Sequence analysis of the on-target site. Both Cas9 and Cas9 nickases induced precise genome editing at the PAM sequence. The numbers of observed sequences over the numbers of total sequenced strains are shown. The error bars show SEM based on more than three independent measurements. P values were determined by comparing between each sample and dead Cas9 control, based on Tukey's test. * $P < 0.05$, ** $P < 0.01$.

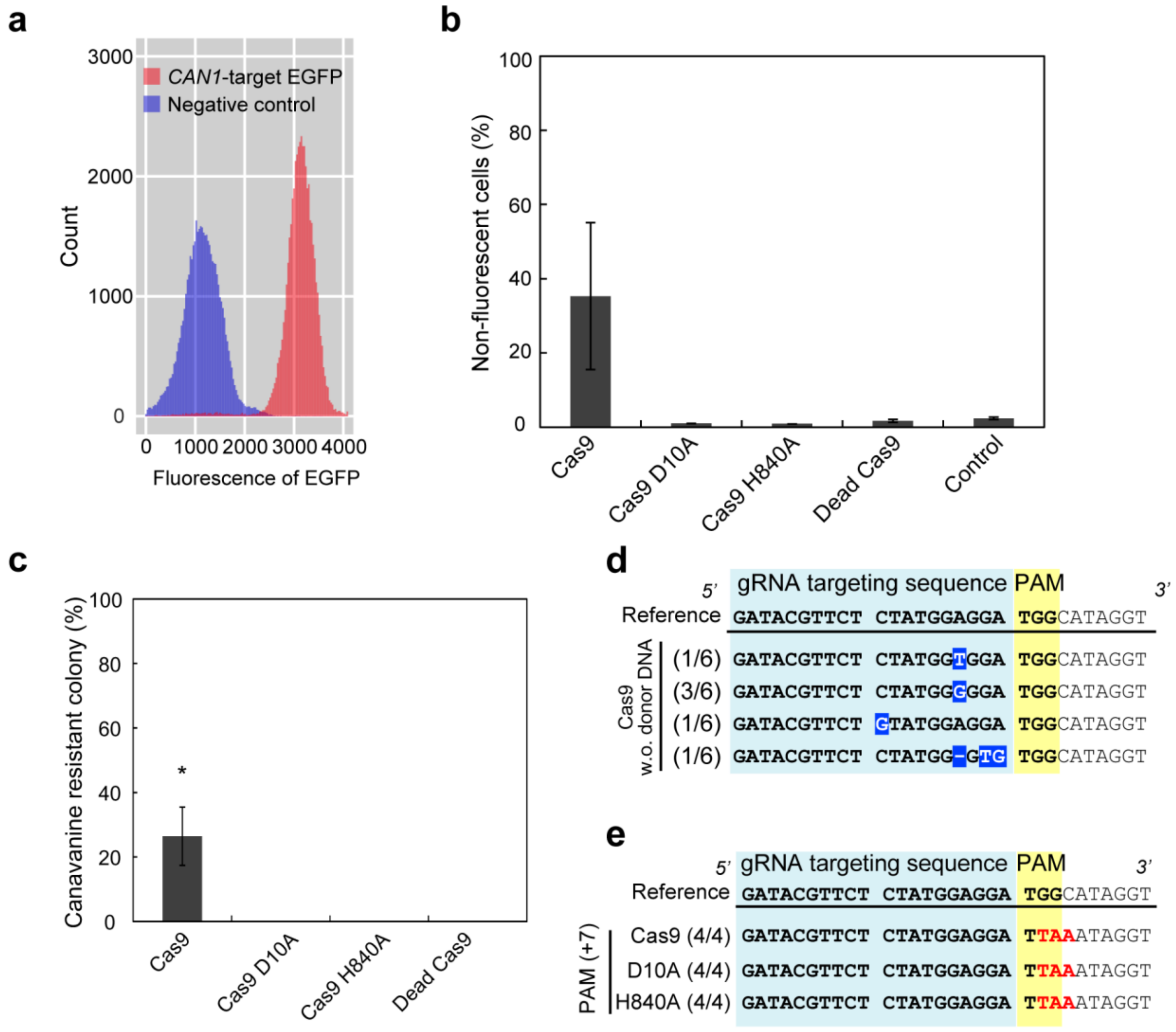

Supplementary Figure S5 Mutant construction by the CRISPR Nickase system coupled with GRC.

(a) Scheme of five-day construction process. DNA fragments with more than 30 bp homology at each end were PCR-amplified from a plasmid template or directly from yeast cells, and the fragments were introduced into yeast cells by transformation. Transformants were grown on selective plate medium (two days) and cultivated in selective medium from an OD600 of 10^{-5} (approximately1.1 × 10^{3} cells) for 48 h (two days). Cells were spread on non-selective palate medium and sequences were analyzed (one day). Mutants can be constructed in a total of five days. (b, c) Sequence analysis corresponding to Fig. 5c, d, respectively. Introduced stop codons (red). The numbers of observed sequences over the numbers of total sequenced strains are shown.

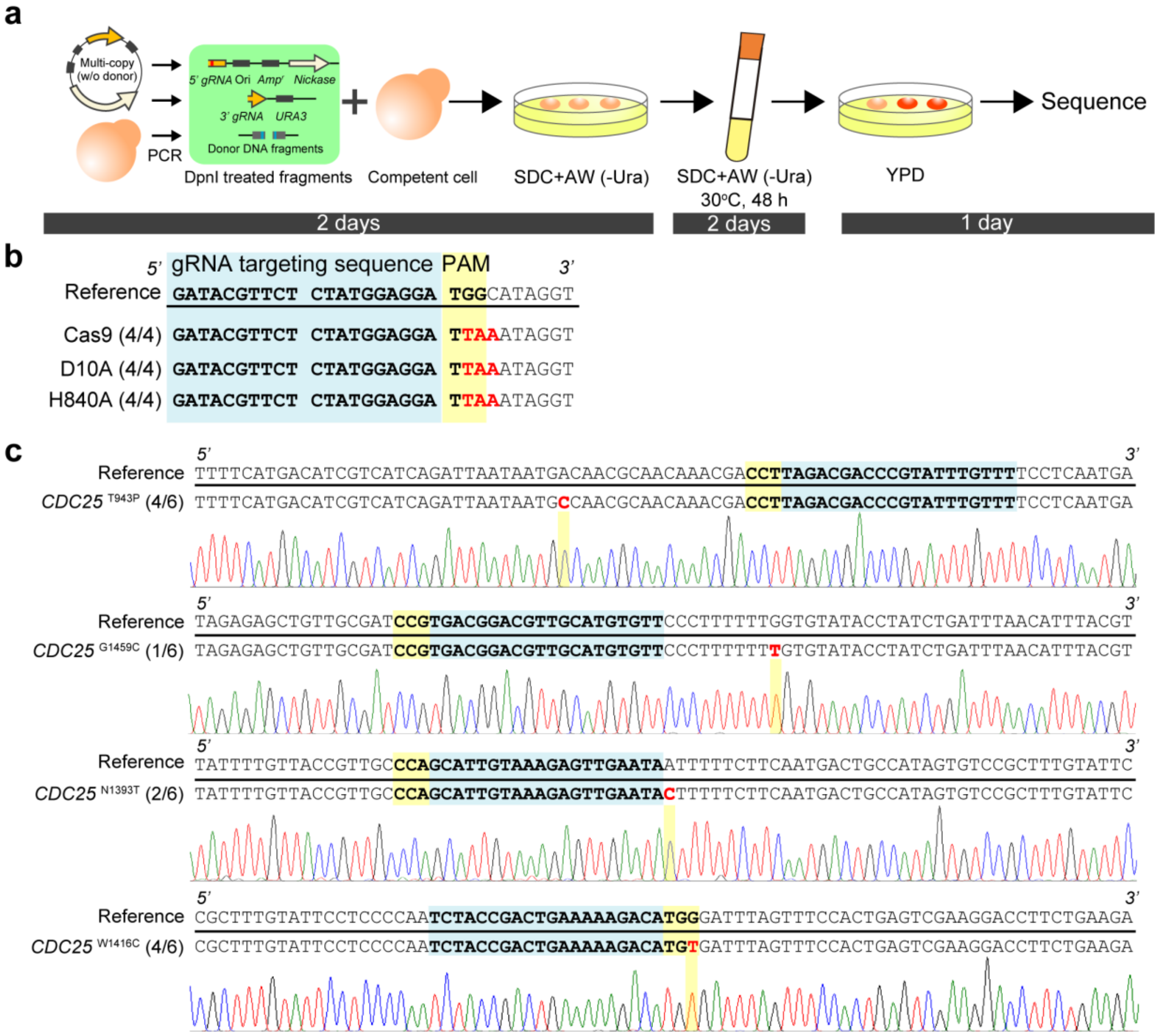

Supplement: Supplementary file 1 — Supporting Information [file 41598_2017_2013_MOESM1_ESM.pdf]
